# Exploring public attitudes toward live-streaming fitness in China: A sentiment and content analysis of China's social media Weibo

**DOI:** 10.3389/fpubh.2022.1027694

**Published:** 2022-11-03

**Authors:** Rui Tian, Ruheng Yin, Feng Gan

**Affiliations:** School of Art, Culture and Tourism Industry Think Tank Chinese Art Evaluation Institute, Southeast University, Nanjing, China

**Keywords:** live-streaming fitness, health communication, Weibo posts, BiLSTM-CNN, STM

## Abstract

**Objectives:**

Live-streaming fitness is perceived by the Chinese government as an invaluable means to reduce the prevalence of physical inactivity amid the COVID-19 pandemic. This study aims to investigate the public altitudes of the Chinese people toward live-streaming fitness and provide future health communication strategies on the public promotion of live-streaming fitness accordingly.

**Methods:**

This study collected live-streaming fitness-related microblog posts from July 2021 to June 2022 in Weibo, the Chinese equivalent to Twitter. We used the BiLSTM-CNN model to carry out the sentiment analysis, and the structured topic modeling (STM) method to conduct content analysis.

**Results:**

This study extracted 114,397 live-streaming fitness-related Weibo posts. Over 80% of the Weibo posts were positive during the period of the study, and over 85% were positive in half of the period. This study finds 8 topics through content analysis, which are fitness during quarantine; cost reduction; online community; celebrity effect; Industry; fitness injuries; live commerce and Zero Covid strategy.

**Conclusions:**

It is discovered that the public attitudes toward live-streaming fitness were largely positive. Topics related to celebrity effect (5–11%), fitness injuries (8–16%), live commerce (5–9%) and Zero Covid strategy (16–26%) showed upward trends in negative views of the Chinese people. Specific health communication strategy suggestions are given to target each of the negative topics.

## Introduction

China has a burgeoning marketplace of live-streaming fitness. In 2021, TikTok, China's leading live-streaming site, reported that the number of fitness live-streamers rose 39% year-on-year, the followers of fitness live-streamers rose 208% year-on-year, and the revenue of fitness live-streamers increased 141% year-on-year (1). Such rapid growth in part stems from the vigorous promotion of the Chinese government. The Chinese government perceives live-streaming fitness as a significant part of China's campaign to reduce the prevalence of physical inactivity, which is a major public health issue in China.

Physical inactivity has been well established as one of the leading factors for non-communicable diseases such as cardiovascular disease, hypertension, diabetes mellitus, obesity, and mental illness (2), which account for about 82% of China's disease burden (3). Moreover, as the COVID-19 pandemic has been causing huge losses both in China's economy and public health, recent studies found that people who are insufficiently physically active are more likely to become seriously ill from COVID-19 than those who actively conduct physical activity (4).

The State Council introduced China's National Fitness Plan in 2010 (5), which is a comprehensive program to tackle the issue of physical inactivity in China (6). The plan has been updated every 5 years since then. Under the auspices of the central government, the prevalence of physical inactivity has decreased. In 2020, the proportion of people who regularly participate in physical exercise in China has reached 37.2% (7). However, the rampant COVID-19 pandemic has abruptly put a damper on the booming trend of China's National Fitness Plan. Further, China's Zero Covid strategy results in the repeated shutdown of parks and gyms, which are two of the main venues for Chinese people to conduct physical activities (8). In response to the ongoing damage the COVID-19 pandemic caused on the reduction of the prevalence of physical inactivity, National Fitness Plan (2021–2025) came into force in August 2021, stipulating that the Chinese government would help facilitate the development of live-streaming fitness in the next 5 years (9).

With the vigorous national promotion, live-streaming fitness grows by leaps and bounds in China. Subsequently, its success has been widely reported (10), and its usefulness and effectiveness have been well analyzed (11). However, the public attitudes toward live-streaming fitness are yet to be sufficiently investigated to fully understand where further improvement is needed. Attitude refers to an individual evaluation tendency to a target object composed of cognition (opinion, belief, knowledge, expectation, etc.) and behavior tendencies (12). Timely and accurate grasp of people's attitudes can be valuable for governments to efficiently develop appropriate strategies to better promote public health policies (13). For this reason, the objective of this study is to fine-tune the analysis by focusing more on the public attitudes of the Chinese people toward live-streaming fitness and hopefully be able to provide health communication advice on the public promotion of live-streaming fitness accordingly.

Public attitudes are usually examined using surveys. However, survey is a very time-consuming research method, and the data collection is often biased due to low response rates (usually well below 10%) (14, 15). To avoid any ineffective or futile issues arising from surveys of this description, scholars are coming to take advantage of the vast amount of data available on the internet as an alternative method. Moreover, the upside of this new method is that online data often unlocks information supporting new insights, particularly in the field of public health. For example, Nobutoshi et al. used the largest Japanese Q&A bulletin board service data to analyze public concerns about influenza vaccinations (16); Shah et al. collected data from physician rating websites (PRWS) to investigate people's attitudes toward various health problems (17), and many more.

Social media has been widely used as data source for public health studies in recent years (18). In comparison with mass media (including online media), social media reaches a wide range of the general public and produces unprecedented user-generated content (19). This study has used Weibo, the Chinese equivalent to Twitter as the data source to explore the public attitudes in China toward live-streaming fitness. As the most open and extensive social media site in China (20), Weibo had 573 million monthly active users and 248 million daily active users by the third quarter of 2021 (21), and, as such, it has been used extensively to capture the collective attitudes of the Chinese people on public health matters. For example, Gao et al. explored changes in the public attitudes toward domestic COVID-19 vaccination after China's vaccines were approved (22); Zhang et al. analyzed the public perception of haze weather (23); Jiang et al. examined public discussions on agricultural product safety incidents (24).

Our research objective is three-fold: (1) to examine the general sentiment of the Chinese people toward live-streaming fitness, (2) to analyze their (dis)satisfaction and (3) to come up with appropriate health communication strategy suggestions for the Chinese government to further promote live-streaming fitness in China. To achieve the above objective, the research questions are therefore framed as follows:

RQ1: What was the overall Weibo activity on live-streaming fitness during the period of the study?

RQ2: What was the general sentiment of Chinese people toward live-streaming fitness during the period of the study?

RQ3: What health communication strategies can be drawn to further promote live-streaming fitness?

This paper makes two major contributions to the present literature. First, this study focused on the (dis)satisfaction of the Chinese people in live-streaming fitness, which was somehow overlooked by the previous literature. In addition, this study has used natural language processing (NLP) techniques to conduct data collection and analysis while the previous literature mainly used conventional research methods such as questionnaires.

**Figure 1:**
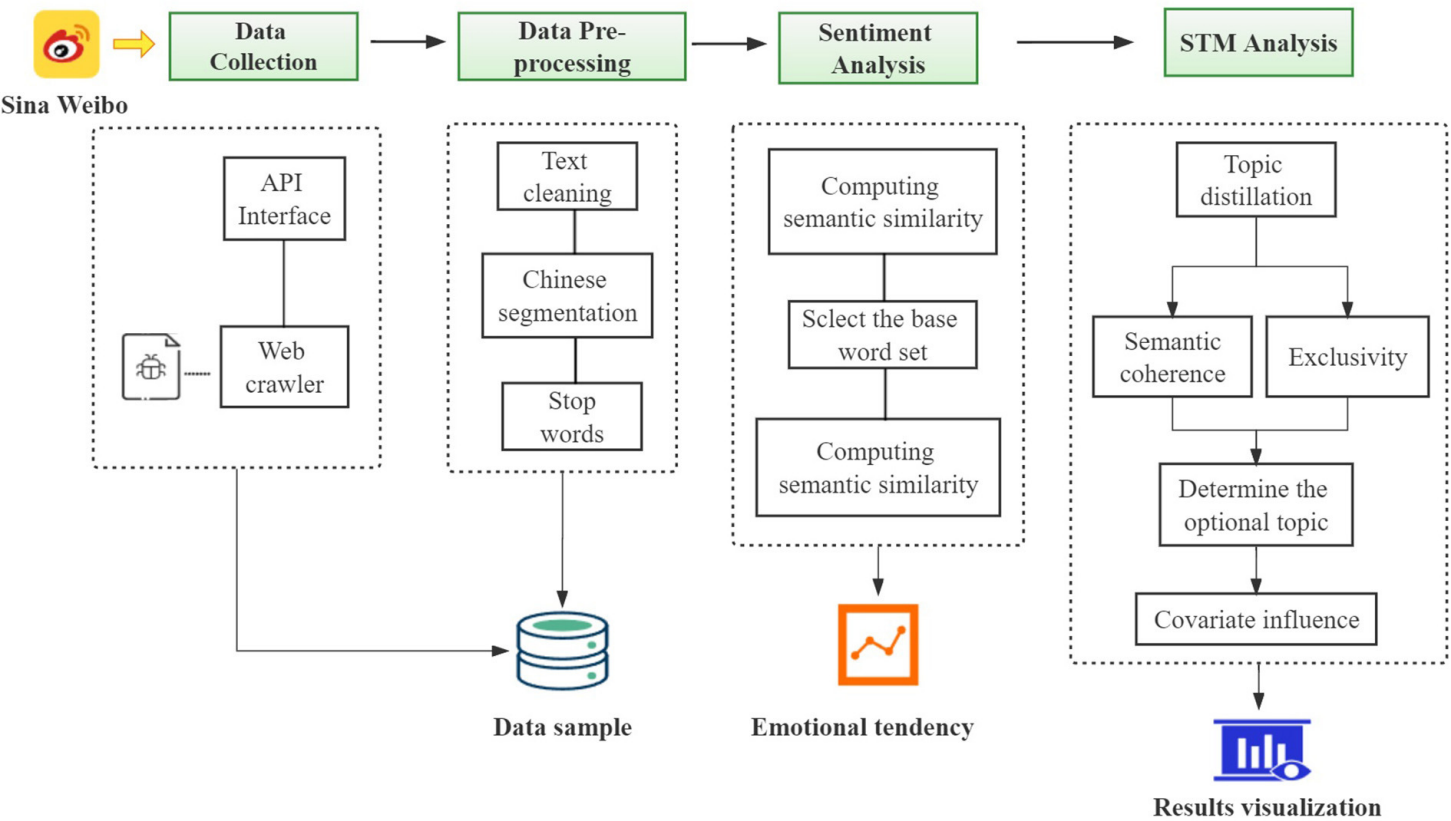
The framework of this study.

Figure 1 shows the research framework of this study. The rest of the paper is organized in three sections. The immediate follow-up section presents the methodology applied in this study, where the process of data collection and processing, sentiment analysis, and content analysis are described. Then, the section of results, which is followed by that of discussions where all the results are thoroughly discussed. At the end of the paper is the final section of conclusions and recommendations for future research are given.

## Methodology

### Data collection and processing

The data collection and processing steps in this study are as follows. First, we used Weibo public streaming application programming interface (API) to extract Weibo posts. The API is provided in Weibo's open platform. Weibo continuously updates its API interface to the needs of users to help users collect data (25). However, Weibo's API has data-fetching restrictions which resulted in insufficient data for the initial return. To address this issue, we wrote a web crawler in Python as a complementary method to further collect data. Next, after checking the number and scope of the textual contents under different keyword searches, we used Live-streaming Fitness as the keywords to extract the research sample. Within the specified time frame (i.e., from July 2021 to June 2022), 174,749 Sina Weibo posts were collected. Last, a second-level cleansing of the dataset was conducted using Pandas, a very effective tool of Python programming to filter out textual data to delete posts that were repeated, meaningless, or consisted of emoji only. The final dataset included 114,397 Weibo posts.

### Sentiment analysis

In order to mine and examine the public attitudes of the Chinese people toward live-streaming fitness, this study conducted both sentiment analysis and content analysis on the collected Weibo posts to monitor the public sentiment and trending opinions of the Chinese people on this fitness model. Sentiment analysis is a desirable method for opinion mining and subjectivity analysis (26), which applies to any textual form of opinion such as microblogs and online reviews (27). Previous studies have demonstrated that sentiment analysis can reveal behavioral and affective trends on public health issues. For example, Zheng et al. tracked the evolution of public sentiment in Wuhan, China, during the first 12 weeks after the identification of COVID-19 on Weibo (28); Salathé et al. analyzed the dynamics of health behavior sentiments on a large online social network (29).

This study adopted a BiLSTM-CNN model to carry out the sentiment analysis. Bidirectional Long-Short Term Memory model (BiLSTM) is an advanced type of Long Short-term Memory model (LSTM). It overcomes LSTM's shortcomings in only memorizing information located at the beginning of a text but not the later parts of the text (30). BiLSTM has proved good results in sentiment analysis, especially for long texts. However, microblog texts are generally short and concise. Therefore, we combined Convolutional Neural Network (CNN) and BiLSTM as a complex model to analyze the sentiment orientation of short texts. CNN is highly effective in recognizing short texts such as Weibo posts (31). The combination of CNN and BiLSTM can capture long-range dependencies and contextual semantic features of the texts, and thus significantly improve the recognition performance of the model. Three steps were followed in this analysis. (1) Each Weibo post was perceived as a unit of analysis (32); (2) By dividing the Weibo posts into positive, negative, and neutral categories, each Weibo post was tagged with a numerical sentiment value from −1 to 1. Values between 0 and 1 indicate positive, and positivity increased with the sentiment value. Values at 0 indicate neutral. Values between −1 to 0 indicate negative, and negativity decreased with the sentiment value; (3) Testing the accuracy of the BiLSTM-CNN model and ensuring the accuracy is over 85%.

### Content analysis

Sentiment analysis is a rather coarse-grained analysis as it cannot address the semantic dimension of public attitudes as expressed in the textual data (33). For this reason, this study also used content analysis to conduct research in a more fine-grained fashion. There are a variety of approaches in terms of conducting content analysis on textual data, such as Word Cloud (34), semantic network (35) and latent Dirichlet allocation (LDA) (36). LDA is one of the most commonly used content analysis approaches when it comes to topic modeling (37), especially in the medical and public health fields (38, 39). It uses a bag-of-words model that treats each textual content as a term-frequency vector, through which textual information is transformed into numerical content that can be easily modeled by computers. Although LDA is widely used by scholars, it is not suitable for this study as it is not able to model topic prevalence using document-level covariates (40). Therefore, this study uses structured topic modeling (STM) instead.

Similar to LDA, STM also assumes that a corpus is a bag of words, and the topics of a text can be measured by the frequency of occurrence of words. In the STM model, each document contains multiple topics, and each topic contains a series of high-frequency words that help define the nature of the topic. Compared to LDA, STM enables the possibility of considering metadata associated with the text using document-level covariates (41) so that researchers can quantitatively analyze the relationship between covariates and topics. Thus, STM is a more suitable method for this study because it allows us to examine the research questions using covariates such as public sentiments and time frame. In recent years, STM is gaining momentum in the academic field. For example, He et al. explored the hidden voice of drug consumers from web-based drug reviews (42); and Pirri et al. examined the public attitudes toward systemic lupus erythematosus (SLE) from Twitter feeds during World Lupus Day19.

**Figure 2:**
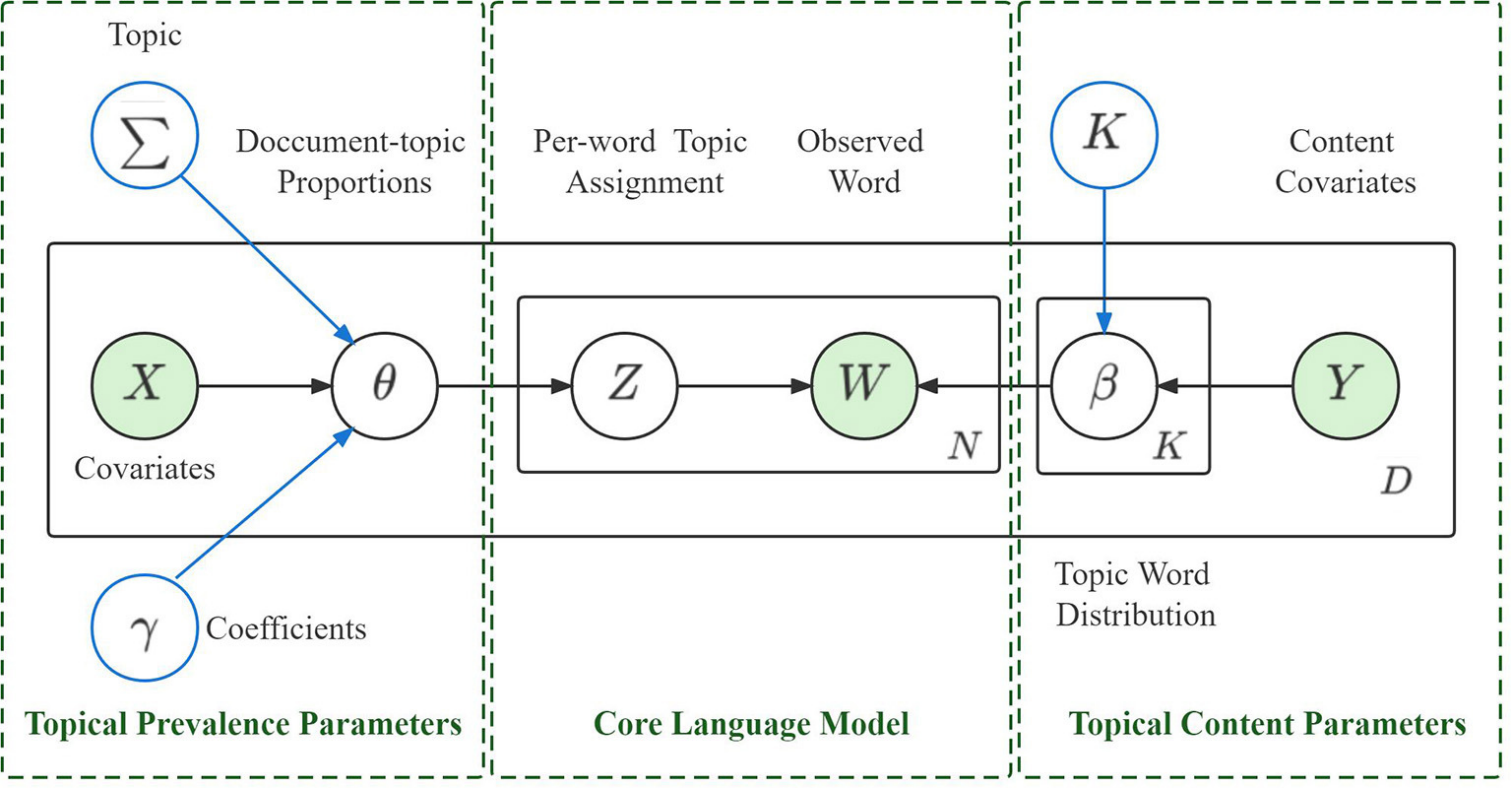
Plate diagram of structural topic model (STM).

The successful construction of a STM model requires the sample size corresponding to the covariates to be as consistent as possible (41). Unbalanced samples would affect the performance of the model (43). Therefore, in this study, we filtered the sample posts to balance the sentiment polarity as the the number of positive Weibo posts significantly exceeded the number of negative Weibo posts. Specifically, we randomly selected the same number of positive and negative Weibo posts. Ultimately, a total of 37,467 Weibo posts were used to build the STM model. Three steps were followed in this analysis. (1) Using Word2Vec to convert Chinese texts into word vectors. (2) Defining the optimal number (k) of the topic, given that each topic consists of five terms with the highest (beta) β probabilities that represented the topic content (Y) (44). (3) Using sentiment polarity and time as two covariates (X) to estimate their effects on topic prevalence. (4) The STM model is parameterized by document specific covariates X and Y. Figure 2 represents the model diagram of the STM in this study.

## Results

### General description

**Figure 3:**
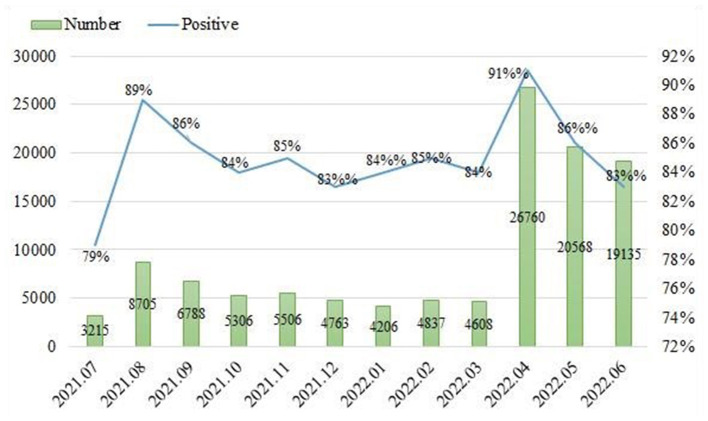
Changes in the number of Weibo posts and the proportion of people's positive emotions.

From the dataset composed of 114,397 Weibo posts, the general picture of the evolution of the popularity of live-streaming fitness among Chinese netizens was drawn (Figure 3). In general, the number of Weibo posts related to live-streaming fitness fluctuated over the months, with two mutation points in August 2021 and April 2022, respectively. The result showed that public interest in the prize was first ignited in August 2021 when the Chinese government introduced the newest version of the National Fitness Plan stipulating that live-streaming fitness will be an integral part of China's national fitness campaign in the next 5 years (2021–2025). In that particular month, a total of 8705 Weibo posts were captured in that month, clocking a marked increase by nearly 5500 increase over the previous month. The second mutation point happened in April 2022, which peaked through the period of the study at 26760 Weibo posts, representing a five-fold leap over the previous month. This took place in the month none other than when Shanghai was under complete lockdown. Over 20 million people of this arguably China's most modernized megacity were quarantined at home. Liu Genghong, a Taiwanese singer who moved to Shanghai to become a fitness live-streamer earlier in 2021 garnered the attention of the entire nation. Millions of people who were trapped at home followed his channel on TikTok and took his live-streaming fitness classes. From April 15 to April 21, the single-day growth of Liu Genghong's TikTok followers continued to rise, and the growth reached its peak on April 21, with a single-day increase of 9.119 million fans. As of May 17, Liu Genghong's TikTok followers have exceeded 67 million, whereas the number was only 3 million 45 days ago.

### Sentiment analysis

Sentiment analysis was conducted on the collected Weibo posts. The sentiment data were reorganized to show the evolution of the public sentiment trend over the given period. Figure 3 examined and visualized variations in the number of Weibo posts and the proportion of people's positive emotions from July 2021 to June 2022. This study originally planned to use trichotomous (positive, neutral, negative) classification. However, during the labeling process, we found that most of the Weibo posts fell into the positive and negative categories while very few Weibo posts (<5%) could be identified as neutral. Therefore, this study ultimately chose dichotomous (positive (non-negative, including neutral) and negative) classification as the small sample size could affects the overall fitting effect of the machine learning model. Overall, the public attitudes in China toward the new fitness model were largely positive. Over 80% of the Weibo posts were positive during the period of the study, and over 85% were positive in half of the period. Meanwhile, around 16% of the Weibo posts were negative. An observable degree of public sentiment fluctuation was demonstrated from the shift in the percentages of positive and negative Weibo posts over the 12-month study period. As shown in Figure 3, in certain months (i.e., August 2021 and April 2022), the Chinese public perceived live-streaming fitness significantly positive, yet in other months (i.e., October 2021 and June 2022), public perceptions were rather negative compared to the average sentiment.

### Content analysis

The premise of conducting STM is to define the optimal number of topics. The topic number is one of the most essential parameters of the STM, which, in practical applications, is usually defined to obtain the most substantive interpretation of the outcomes rather than the maximization of the topics (40). In this study, the semantic coherence of the words (45) and the exclusivity of the topics are two metrics that help determine the outcome of the optimal number of topics (46). The semantic coherence metric measures the most probable words in a specified topic that occur together. When this simultaneous occurrence is frequent, semantic coherence will thus reach its maximization. The exclusivity metric indicates that the most probable words will not appear in any other topics rather than the specified topic at the same time. Once with the semantic coherence and exclusivity of the outcomes of a series of models with different topic numbers checked, we ultimately adopted the eight-topic model as this model yielded the most semantically coherent and exclusive outcomes in our experiment.

The results of the topic clustering are shown in Table 1. Each row in Table 1 represents a topic that is frequently discussed in Weibo. The first column demonstrates the topic labels, which are not automatically generated (47). Label selection was the moment when researchers defined the nature of topics through the analysis of the top key terms linked to the specified topic (48). In our case, for each topic a specified label was determined using the authors' unanimous judgments through an open discussion with two experts in the field of public health and one expert in the field of sport management. The second column represents the proportion of each topic. The third column lists the five terms that have the highest probability in the specified topic. Topic 1 is about the nature of live-streaming fitness in the pandemic, which is a worthy substitute of outdoor fitness. The terms include Covid, health, lockdown, etc. Topic 2 shows the advantage of live-streaming fitness in reducing cost over outdoor fitness. The terms include economic cost, time cost, consumption, etc. Topic 3 focuses on the online community formed through live-streaming fitness. The terms include communication, quarantine, friendship in cyberspace, etc. Topic 4 addresses the celebrity effect in live-streaming fitness. The terms include Liu Genghong, Jay Chou, celebrity, etc. Topic 5 is about the live-streaming fitness industry that is in the making. The terms include industry, job, internship, etc. Topic 6 focuses on the possible fitness injuries caused by following live-streaming fitness programmes. The terms include knee, soreness, pain, etc. Topic 7 is about the live commerce conducted in the live-streaming fitness. The terms include live commerce, quality, supervision, etc. Topic 8 shows the dissatisfaction voiced against China's Zero Covid policy. The terms include Shanghai, unnecessary, lockdown, etc.

**Table 1:** Topics generated by STM.

**Number**	**Labels**	**Proportion (%)**	**Top words**
1	Fitness during quarantine	21.06	Covid, health, lockdown, quarantine, live-streaming
2	Cost reduction	16.43	Economic cost, time cost, consumption, cost performance, membership
3	Online community	9.08	Community, chat, fitness friends, together, relief
4	Celebrity effect	19.27	Liu Genghong, Jay Chou, celebrity, endorsement, fans
5	Industry	4.50	Industry, job, internship, salary, easy
6	Fitness injuries	10.26	Soreness, pain, knee pain, sprained ankles and low back pain
7	Live commerce	11.37	Live commerce, quality, supervision, tax, experience
8	Zero Covid strategy	8.03	Shanghai, starving, prisoner, lockdown, unnecessary

To better understand the meaning of the topics, we also examined the most representative Weibo posts of each clustered topic. Table 2 shows the topics and the associated Weibo posts.

**Table 2:** Most representative Weibo texts and topic label selection.

**Fitness during quarantine (topic 1):**
“I think live-streaming fitness is very interesting.”; “Because of the pandemic, we are not able to go to the gym. Live-streaming fitness is a good thing.”; “Live-streaming fitness is the new norm of physical activity.”
**Cost reduction (topic 2):**
“Most people simply do not have to spend money and time to go to the gym. Live-streaming fitness is more than enough.”; “I'm not a fitness enthusiast. Bye-bye gym membership fee! Live-streaming fitness is perfect!”; “I don't have time to go to gym everyday. Live-streaming fitness is way better.”
**Online community (topic 3):**
“Live-streaming fitness is such a great way to meet girls! I find it far more efficient than blind dates! ”; “Live-streaming fitness is so different. Millions of people are working out with me. It feels so good!”; “I'm not alone!”
**Celebrity effect (topic 4):**
“So many celebrities are now fitness live streamers!”; ” I'm a huge fan of Chen Yiru. I followed his live-streaming to exercise!”; “Li ruotong is the reason why I do this.”
**Industry (topic 5):**
“Live-streaming fitness is the future of the sports industry”; “I just submitted my resume to Liu Genghong's team.”; “So many job opportunities in livestraming fitness industry right now. How exciting!”
**Fitness injuries (topic 6):**
“I followed Liu Genghong's live streaming yesterday. I could barely get out of bed this morning.”; “I twisted my left ankle last night.”; “Liu Genghong's class is not for me. My knee is killing me!”
**Live commerce (topic 7):**
“Why can't they focus on the things they are good at!”; “They shouldn't do live commerce during the break. It ruined the fitness experience.”;” Tiktok needs to do something. I can't believe how many breaks they had last night to do live commerce. They are ruining live-streaming fitness. ”
**Zero Covid strategy (topic 8):**
“Shanghai is under complete lockdown. I can't do anything rather than following Liu Genghong's fitness live-streaming. This is so depressing. ”; “The government is just using Liu Genghong and live-streaming fitness to shun the blame.”; “I'm starving. How can I participate in live-streaming fitness when there is no food!”.

**Figure 4:**
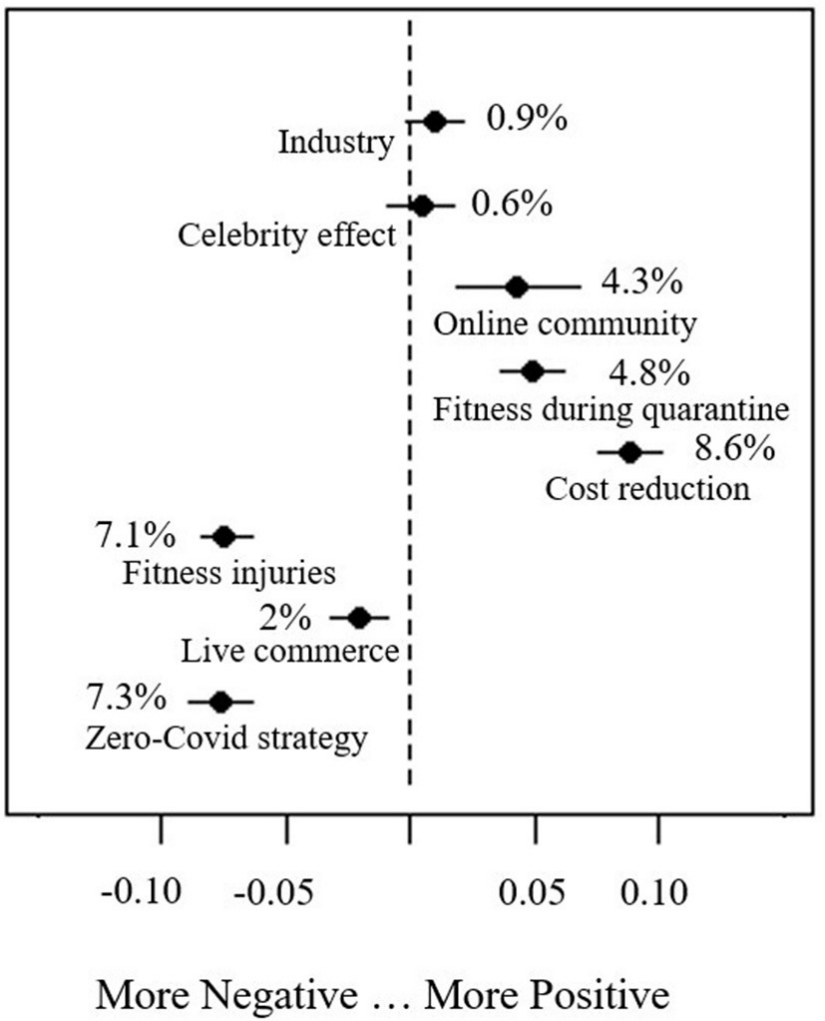
Change in topic prevalence based on review extremity (positive vs. negative).

From the topic modeling results, latent themes behind the Weibo discussions emerged, underlying a hidden structure that reflected the interests and concerns of the Chinese people toward live-streaming fitness. As previously mentioned, to further promote live-streaming fitness in China, we must understand the satisfaction and dissatisfaction that Chinese people have on live-streaming fitness. Only then can we come up with efficient strategies accordingly. To this end, this study added public sentiment as a covariate in the STM model. In other words, a topic will be identified as negative if the proportion of this topic in the negative Weibo posts is noticeably larger than it in the positive ones and vice versa (49). The results were shown in Figure 4. The points represent the mean values of the estimated differences, and the bars represent the 95% confidence intervals of the differences. The dotted line in the middle indicates zero effect. The right direction of the dotted line represents the positive effect, and the left direction of the dotted line represents the negative effect. Take topic “fitness during quarantine” as an example, the proportion of positive Weibo posts of this topic is higher than that of negative reviews by 4.8%, and the difference exists in 3.6–6.4% confidence interval. Therefore, “fitness under quarantine” can be identified as a positive topic. As shown in Figure 4, topics related to fitness under quarantine, reduction of cost, online community, celebrity effect and industry are positive topics, while topics focused on injuries, live commerce and policy dissatisfaction are negative topics.

**Figure 5:**
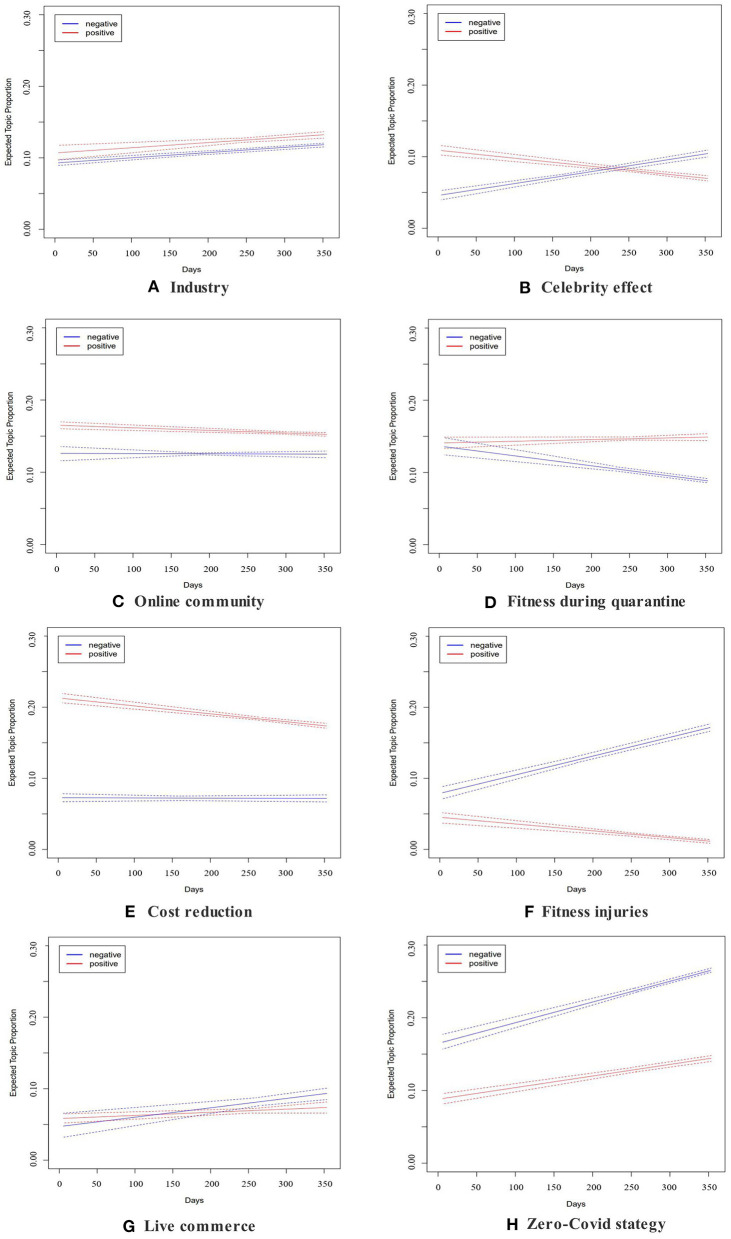
**(A–H)** Moderating effects of time.

Given that live-streaming fitness is an emerging fitness model in China, which is subject to change over time, the satisfaction and dissatisfaction of the Chinese public toward live-streaming fitness could change accordingly. Therefore, by tracking the most frequent topics overtime, we could able to offer a more detailed analysis on the evolution of the public sentiment toward live-streaming fitness. To achieve this research goal, we have implemented an interaction term of time and public sentiment into the STM model. Figure 5 demonstrates the evolution trend of the topic proportion through the period of study. The x-axis represents time, which uses days as the unit. The y-axis represents the topic proportion. The blue and red lines represent the proportions of negative and positive Weibo posts, and the dashed line represents the 95% confidence interval.

To locate the dissatisfaction of the Chinese people toward live-streaming fitness, this study focused more on the changes in negative sentiments. Figures 5A–E shows the evolution of the negative sentiments in positive topics overtime. Except for celebrity effect (topic 4), which increased from 5 to 11%, none of the positive topics showed significant upward trends in the prevalence in negative Weibo posts during the study period. In particular, fitness during quarantine (topic 1) reported a noticeable decrease from 13 to 8%. By comparison, cost reduction (topic 2), online community (topic 3), and industry (topic 5) maintained their respective proportions in negative Weibo posts. The fluctuations in negative topic proportions of these three topics are around 1%.

Figures 5F–H represents the changes in the negative sentiments in negative topics during the study period. The negative sentiment toward these topics continued to rise throughout the study period. Among them, Zero Covid strategy (topic 8) showed a startling increase of 10% in the prevalence in negative Weibo posts (16–26%). Similarly, fitness injuries (topic 6) reported a two-fold increase in the prevalence in negative Weibo posts (8–16%) during the study period. In comparison, the upward trend of live commerce (topic 7) in the prevalence in negative Weibo posts was relatively moderate (5–9%).

## Discussions

To answer the first research question is about the overall live-streaming fitness-related Weibo activity, the result suggests that public interest in live-streaming fitness was ignited twice over the last 12 months. The first one was in August 2021 when the Chinese government officially acknowledged the significance of live-streaming fitness. The corresponding surge in Weibo discussions could come from the vast amount of media spotlight on the acknowledgment at that time as mass media often rushes in to promote the communication and understanding of public policy (50). The other one was in April 2022 when Liu Genghong, a celebrity set off the craze for live-streaming fitness in Shanghai and then the entire nation. This result could be due to the celebrity effect. Celebrity publicity often raises the public interest in public health issues. A good example is that when Angelina Jolie detailed her decision to undergo a double mastectomy to prevent herself from breast cancer in New York Times column, the daily BRCA test rates increased by 64% in the next 15 business days (51).

With regards to the second research question on the public sentiment of Chinese people toward live-streaming fitness, our findings showed that the majority of the Chinese public held positive attitudes (86.2%) about live-streaming fitness, as compared to the negative (13.8%) opinion holders. In addition, the two incidents that sparked the nationwide interest in live-streaming fitness also affected the public attitudes according to the public sentiment proportions. The first incident increased the positive view of the Chinese people (79–89%), which again proved the significant impact mass media has exerted on promoting public policies. Meanwhile, the second incident caused a decrease on the proportions of the positive Weibo posts. The sudden success of Liu Genghong put live-streaming fitness in the national spotlight for scrutiny. Unlike the previous time when people focused solely on the positive information of live-streaming fitness fed by the government through mass media, various problems of this new fitness model were exposed in the media scrutiny and were noticed by the Chinese public, which subsequently lowered the proportions of the positive attitudes.

To further examine this research question, this study also looked into the evolution of the negative sentiment toward the eight topics generated by the STM (42). In doing so, we can pinpoint Chinese people's concerns toward live-streaming fitness and thus propose more appropriate health communication strategies to reverse any negative trend accordingly. As shown in Figure 5, four topics showed an observable upward trend in negative Weibo posts proportions during the study period. Among them, Fitness injuries (topic 6) reported significant surge in the prevalence in negative Weibo posts (8–16%). There have been numerous reports that people were injured due to their participation in live-streaming fitness (52), making this topic one of the major concerns of the Chinese people toward live-streaming fitness. The topic prevalence of celebrity effect (topic 4) in negative Weibo posts increased from 5 to 11%, showing that the Chinese people were losing interest in Liu Genghong and his routines, which also reflects in the decline of his viewers, and a demand of something new in the particular area. Live commerce (topic 7) also demonstrated an upward trend in the prevalence in negative Weibo posts (5% to 9%). Fitness live-streamers have been striving to incorporate live commerce to the fitness classes (53), which appears to heap criticism among the Chinese people. Zero Coivd strategy (topic 8) reported the biggest surge in the prevalence in negative Weibo posts (16–26%). However, after looking into the top terms linked to the topic and the most represented Weibo posts, we found that the dissatisfaction exposed in this topic was not directed at the live-streaming fitness but at China's Zero Covid policy.

From the analysis of the data around the first and the second research questions, a clear answer to the third research question about drawing health communication strategies to further promote live-streaming fitness unfolds itself. We propose the following health communication strategy-related suggestions to address the major concerns the Chinese people have against live-streaming fitness. First, in response to the potential fitness injuries live-streaming fitness could inflict upon its participants, the government should enhance the promotion of sports health information in the media to prevent people from injuring themselves due to lack of knowledge in such field. Second, the government should encourage more celebrities to endorse live-streaming fitness. Celebrities can efficiently promote live-streaming fitness through participating and advocating on social media, just as Liu Genghong did in April 2022. Third, the government should underscore the importance of separating live fitness and live commerce and require fitness live streamers to leave live commerce at the end of fitness sessions, thus avoiding interference with the fitness experience. Last, the results reflected the public anguish over China's strict policies on quarantines and lockdowns. Since the government showed no sign of changing course in this matter, it should step up its efforts in public education and emphasize on the great potential physical activity for the prevention and treatment of anxiety and anxiety and depression (54).

## Conclusions

A couple of limitations of this study need to be mentioned. First, this study collected data from only one social media site. Many individuals may have also dis-cussed about live-streaming fitness within online forums, Wechat groups, and other social media platforms. Another limitation of using social media posts as research data is that posts with extreme sentiments were often removed by the social media plat-forms. This may have reduced the overall percentage of a particular emotion in our samples.

Notwithstanding these limitations, this study provides a clear indication of the public attitudes toward live-streaming fitness in China. This study used Weibo, China's leading social media site, as the data source to examine the attitudes of Chinese people. Data were extracted via Weibo public streaming API and a web crawler we programmed in python. In doing so, we are able to build a huge dataset that is extremely hard to achieve through traditional research methods such as surveys. A BiLSTM-CNN based sentiment analysis was employed to broadly evaluate public attitudes by measuring the degrees of public sentiment polarity (positive and negative). For a profound understanding of the data, sample posts were further examined by using STM to extract the main topics of Weibo discussions and then analyze the public attitudes from a topic perspective. The results show that Chinese people, in general, hold a positive attitude toward live-streaming fitness. However, the dissatisfaction of the Chinese public over topics such as fitness injuries, celebrity effect, live commerce and COVID-19 policies were also identified. Suggestions on health communication strategy were given accordingly.

## Data availability statement

The raw data supporting the conclusions of this article will be made available by the authors, without undue reservation.

## Author contributions

Conceptualization, methodology, software, validation, formal analysis, writing—original draft preparation, and visualization: RT and RY. Writing—review and editing: RT, RY, and FG. Supervision and project administration: FG. All authors have read and agreed to the published version of the manuscript.

## Funding

This research was supported by the National Social Science Foundation (21ZD11) and Jiangsu Provincial University Philosophy and Social Science Research Fund (2020SJZDA078).

## Conflict of interest

The authors declare that the research was conducted in the absence of any commercial or financial relationships that could be construed as a potential conflict of interest.

## Publisher's note

All claims expressed in this article are solely those of the authors and do not necessarily represent those of their affiliated organizations, or those of the publisher, the editors and the reviewers. Any product that may be evaluated in this article, or claim that may be made by its manufacturer, is not guaranteed or endorsed by the publisher.
