# Supplementary material for: Exploring public attitudes toward live-streaming fitness in China: A sentiment and content analysis of China's social media Weibo
Source: Front Public Health. 2022 Nov 3;10:1027694. doi: 10.3389/fpubh.2022.1027694 (PMC9669485; doi:10.3389/fpubh.2022.1027694)
Supplement: Supplementary file 1 [file Table_1.DOCX]

**Paper ID:** 1027694

**Title:** Exploring Public Attitudes towards Live-streaming Fitness in China: A Sentiment and Content Analysis of China’s Social Media Weibo

**The details of the model training are as follows:**

This study built a neural network model to extract key features from Weibo texts embeddings. Table 1 shows the model parameters.

Table 1. Model layers and configurations

| Layer | Description |
| --- | --- |
| Embedding | 1. Embedding(13838, 300, padding_idx=1) |
| Convolutional NN | 1. Conv2d(1, 300), kernel_size=(3, 300), stride=(1, 1), padding=(1, 0), 2. Max_pool1d(), 3. Conv2d(1, 300, kernel_size=(4, 300), stride=(1, 1), padding=(2, 0), 4. Max_pool1d(), 5. Conv2d(1, 300, kernel_size=(5, 300), stride=(1, 1), padding=(2, 0), 6. Max_pool1d() |
| Dropout | 1. Dropout(p=0.75, in_place=False) |
| Bidirectional LSTM | 1. LSTM(300, 300, 2, dropout=0.75, bidirectional=True, bias=True) |
| Linear | 1. Linear(in_features=1500,out_features=750, bias=True) 2. Linear(in_features=750, out_features=2, bias=True) |

Figure 1 presents the network of the model.


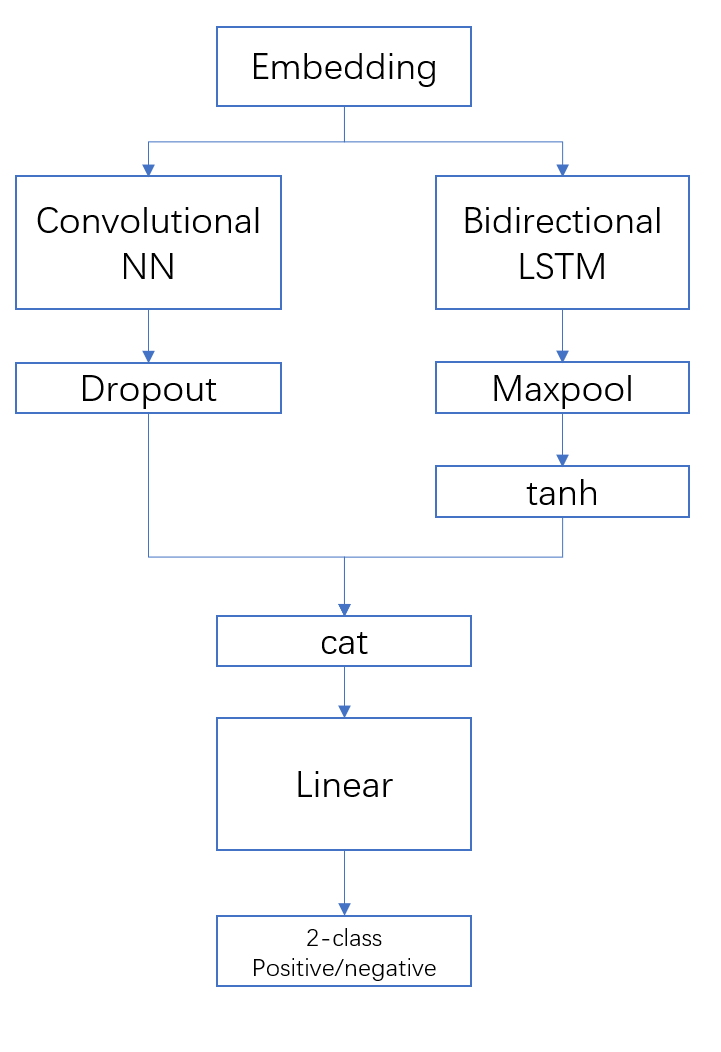


Figure 1. The network of the model

The training hyperparameters are shown in Table 2.

Table 2 Training hyperparameters

| Batch size | 16 |
| --- | --- |
| #CNN kernel | 300 |
| #CNN layers | 3 |
| LSTM hidden dim | 300 |
| #LSTM layers | 2 |
| Learning rate | 0.001 |
| Weight_decay | 1e-8 |
| Optim_momentum_value | 0.9 |
| #epoch | 500 |
| Eval_interval | 200 |
| Save_interval | 200 |
